# Lysosomal-Associated Transmembrane Protein 5 (LAPTM5) Is a Molecular Partner of CD1e

**DOI:** 10.1371/journal.pone.0042634

**Published:** 2012-08-03

**Authors:** Catherine Angénieux, François Waharte, Alexandre Gidon, François Signorino-Gelo, Virginie Wurtz, Rim Hojeij, Fabienne Proamer, Christian Gachet, Alain Van Dorsselaer, Daniel Hanau, Jean Salamero, Henri de la Salle

**Affiliations:** 1 Institut National de la Santé et de la Recherche Médicale, Unité Mixte de Recherche S725, Strasbourg, France; 2 Etablissement Français du Sang-Alsace, Strasbourg, France; 3 Université de Strasbourg, Strasbourg, France; 4 Cell and Tissue Imaging Facility, Unité Mixte de Recherche 144, CNRS-Institut Curie Section de Recherche, Paris, France; 5 Molecular mechanisms of intracellular transport, Unité Mixte de Recherche 144, CNRS-Institut Curie Section de Recherche, Paris, France; 6 Institut National de la Santé et de la Recherche Médicale, Unité Mixte de Recherche S949, Strasbourg, France; 7 Centre National de la Recherche Scientifique, Unité Mixte de Recherche 7178, Strasbourg F-67037, France; Karolinska Institutet, Sweden

## Abstract

The CD1e protein participates in the presentation of lipid antigens in dendritic cells. Its transmembrane precursor is transported to lysosomes where it is cleaved into an active soluble form. In the presence of bafilomycin, which inhibits vacuolar ATPase and consequently the acidification of endosomal compartments, CD1e associates with a 27 kD protein. In this work, we identified this molecular partner as LAPTM5. The latter protein and CD1e colocalize in trans-Golgi and late endosomal compartments. The quantity of LAPTM5/CD1e complexes increases when the cells are treated with bafilomycin, probably due to the protection of LAPTM5 from lysosomal proteases. Moreover, we could demonstrate that LAPTM5/CD1e association occurs under physiological conditions. Although LAPTM5 was previously shown to act as a platform recruiting ubiquitin ligases and facilitating the transport of receptors to lysosomes, we found no evidence that LATPM5 controls either CD1e ubiquitination or the generation of soluble lysosomal CD1e proteins. Notwithstanding these last observations, the interaction of LAPTM5 with CD1e and their colocalization in antigen processing compartments both suggest that LAPTM5 might influence the role of CD1e in the presentation of lipid antigens.

## Introduction

The mammalian CD1 proteins form a system of molecules which participate in the presentation of lipid antigens to αβ and γδT cell subsets. The five *CD1* genes present in the human genome are expressed in various cell types including dendritic cells (DCs), the professional antigen presenting cells of the immune system. The *CD1* genes encode proteins which, according to their sequence homologies, patterns of expression and functional attributes, may be divided into three types. The first two are referred to as group 1 (CD1a, CD1b and CD1c) and group 2 (comprising only CD1d), which present lipids. The fifth form, CD1e, appears to belong to another branch in the evolution of mammalian CD1 proteins [1] and characteristically, does not directly present antigens to T cells.

Unlike other CD1 molecules, human CD1e displays an exclusively intracellular localization in DCs (e.g. interstitial DCs or epidermal Langerhans cells) and thymocytes [2]. In immature DCs, the membrane anchored CD1e molecules accumulate in trans-Golgi compartments (TGCs). These molecules are transported to lysosomes where they are cleaved into soluble proteins [2]. Lysosomal soluble CD1e represents the active form and is only found in mature DCs; it assists lysosomal α-mannosidase in the antigenic processing mycobacterial phosphatidylinositol hexamannoside (PIM6) into an antigenic dimannosylated form (PIM2), which is presented by CD1b. In this way, CD1e extends the repertoire of microbial glycolipid T antigens [3]. Moreover, CD1e modulates the presentation of endogenous and exogenous lipid antigens by CD1b, c and d. This results in part from its capacity to accelerate the formation and dissociation of CD1-lipid complexes. Thus, CD1e participates in antigen presentation, not only by shaping the repertoire of available lipid antigens, but also by influencing the generation and persistence of group 1 and group 2 CD1-lipid complexes; i.e., it tunes T cell responses to CD1-restricted lipid antigens in a temporal manner [4].

One aspect of our work is the elucidation of the mechanisms controlling the transport of CD1e proteins. We have shown that several parts of the CD1e cytoplasmic tail influence its cellular distribution, while its ubiquitination facilitates generation of the soluble lysosomal form [5]. In addition, CD1e appears to associate with a 27 kD protein (p27) when it is transported to lysosomes. This association, only documented when acidification of the endosomal pathway was blocked with bafilomycin, an inhibitor of vacuolar ATPase, was observed in immature DCs as well as in transfected M10 cells [2]. The aims of the present work were to identify p27, to determine whether it interacts with CD1e proteins under physiological conditions and to see whether this interaction has consequences for the transport and/or function of CD1e.

## Methods

### Cells, culture media and reagents

HEK293 and HeLa cell lines (CRL-1573 and CCL-2, respectively) were obtained from ATCC (LGC Standards, Molsheim, France). M10 [6] and FO-1 [7] are melanoma cell lines. HeLa, HEK293 and FO-1 cells were grown in Dulbecco culture medium, M10 cells in RPMI 1640, and all were supplemented with 10% fetal calf serum (Invitrogen, Cergy-Pontoise, France). Transfected M10 cells expressing CD1e molecules have been previously described [2]. Monocytes and blood cells were obtained from voluntary healthy donors who routinely give their blood in our institution, EFS-Alsace (regional blood transfusion center), and who agree through a written consent signed each time they give blood cells, that their blood cells can be used for scientific research purposes. DCs were differentiated from elutriated monocytes using IL-4 and GM-CSF [8] and their maturation was induced with 1 µg/mL *E. coli* LPS (Sigma Chemical Co., Saint-Quentin Fallavier, France). When indicated, cells were incubated with 0.1 µM bafilomycin (LC Laboratories, Woburn, MA).

### Mass spectrometric identification of LAPTM5

Preliminary experiments revealed that p27 and the light chain of the anti-CD1e mAb 20.6 displayed similar if not identical electrophoretic mobilities (data not shown). Adherent M10 cells, untransfected or expressing transgenic CD1e (500×10^6^ cells/sample), were incubated for 5 hours in complete culture medium supplemented with bafilomycin. After detachment with Versene (Invitrogen), the cells were centrifuged, washed in PBS and, the membranes were solubilized in 25 mL of lysis buffer containing 1% Triton X-100, 150 mM NaCl, 20 mM Tris (pH 8) and a cocktail of protease inhibitors (Complete Mini, Roche Applied Science, Meylan, France). After a 10 min centrifugation (13,000×g), the cleared lysates were incubated twice with 0.5 mL of protein A-Sepharose for 1 h and centrifuged again. The supernatants were then incubated overnight with 0.2 mL of protein A-Sepharose carrying the immobilized mAb 20.6 (100 µg), after which the matrix was pelleted and extensively washed. Immunoadsorbed proteins were eluted in 100 µL of Laemmli buffer and separated by SDS-PAGE. After staining the gels with Coomassie blue, one mm sections were cut. The gel pieces were washed in 100 µL of 25 mM NH_4_HCO_3_, dehydrated twice in 100 µL of acetonitrile and dried in a SpeedVac evaporator, before reduction (10 mM DTT in 25 mM NH_4_HCO_3_) and alkylation (55 mM iodoacetamide in 25 mM NH_4_HCO_3_).

For tryptic digestion, the gel pieces were resuspended in three gel volumes of trypsin (12.5 ng/µL) freshly diluted in 25 mM NH_4_HCO_3_ and incubated overnight at 35°C. The digested peptides were then extracted from the gel in a buffer containing 25% H_2_O, 70% acetonitrile and 5% HCOOH and analyzed by LC/MS/MS. For nano-HPLC, a CapLC system (Micromass Ltd., Manchester, UK) was used. The samples were concentrated on a precolumn, after which the peptides were separated on a 15 cm×75 µm i.d. column packed with 3 µm 100 Å C18 PepMap (LC-Packings). MS and MS/MS analyses were performed with a Q-TOF 2 hybrid quadrupole/time-of-flight mass spectrometer (Micromass Ltd.) equipped with a Z-spray ion source. LC/MS/MS data were processed automatically using the ProteinLynx Process (Micromass Ltd.) module. Data analysis was performed with Global Server (Micromass Ltd.) and Mascot (Matrix Science Ltd., London, UK) software using the NCBI (National Center for Biotechnology Information) database.

### Cellular RNA extraction and reverse transcription PCR

Total cellular RNA was extracted with an RNeasy Mini extraction kit (Qiagen, Courtaboeuf, France). RNA (2 µg) was transcribed into cDNA using random hexanucleotide primers (Roche Applied Science) and Superscript III reverse transcriptase (Invitrogen) according to the manufacturers' instructions. Amplifications were performed using 100 ng of reverse transcribed RNA, recombinant Taq DNA polymerase (Invitrogen) and the LAPTM5-specific primers (+) 5′-CTGCAAGCTCTCCCAGATGG-3′ and (−) 5′-CACCTTCTGGAGCATCTTGG-3′ (94°C - 45 s, 56°C - 30 s, 72°C - 1 min, 30 cycles). As an internal control, the expression of actin was checked by RT-PCR using the primers: (+) 5′-GACTACCTCATGAAGATCCT-3′ and (−) 5′-ATCCACATCTGCTGGAAGGT-3′.

### Expression vectors and transfected cells

Expression vectors for LAPTM5 and the EYFP-LAPTM5 fusion protein, in pEF-DEST51 and pdEYFP-C1amp respectively, were purchased from imaGenes GmbH (Berlin, Germany). To select stably transfected cell lines, a blasticidine resistance gene (from the pUB vector digestion fragment EcoRV-SmaI, Invitrogen) was inserted into the NaeI restriction site of the latter vector. A V5 tag was added to the C-terminal end of LAPTM5 in the pEF-DEST51 expression vector by PCR-based mutagenesis. The expression vector for the CD1e-mCherry fusion protein has been previously described [5].

Cell lines were transfected using Fugene reagent (Roche Applied Science). Stably transfected M10 cells were selected using 5 µg/mL blasticidine (InvivoGen, Toulouse, France) and/or 500 µg/mL G418 (Invitrogen). Two-fold higher concentrations of these antibiotics were used to select transfected HEK293 cells. Transfected clones expressing EYFP-LAPTM5 fusion molecules were screened for the fluorescence of EYFP. Transfected clones expressing V5-tagged LAPTM5 proteins were selected by immunofluorescence staining of fixed, permeabilized cells using an anti-V5 mAb coupled to Alexa-488 (A-488) (AbD Serotec, Oxford, UK).

### Silencing experiments

Anti-LAPTM5 and anti-GAPDH inducible shRNAmir lentiviral plasmid vectors were purchased from OpenBiosystems (Thermo Fisher Scientific, Illkirch, France). Lentiviral particles were prepared in HEK293 cells and purified by ultracentrifugation as described elsewhere [9]. Transfected M10 cells expressing CD1e were transduced and selected with puromycin. Silencing was induced by treatment with 1 µg/mL doxycycline for 48 h and was checked by RT-PCR, followed by gel electrophoresis of the amplified products and ImageJ analysis. RT-PCR analysis of actin expression was used as the standard.

### Antibodies

The anti-CD1e mAbs VIIC7 and 20.6 have been described previously [2]. These mAbs were chemically coupled to dimethyl pimelimidate (DMP; Fisher Scientific, Illkirch-Graffenstaden, France) on protein A-Sepharose (GE Healthcare, Orsay, France) (20.6-DMP or VIIC7-DMP) as previously described [10].

Rabbit anti-GFP (Invitrogen), mouse anti-GFP (Roche Applied Science), mouse anti-V5 (Invitrogen) and HRP-conjugated mouse anti-ubiquitin antibodies (Santa Cruz) were used for immunoprecipitation and western blotting.

For confocal microscopy immunofluorescence, were used: polyclonal sheep anti-TGN46 Abs (AbD Serotec), polyclonal goat anti-EEA1 Abs (N-19; Santa Cruz Biotechnology, Santa Cruz, CA), unconjugated and FITC-conjugated L243 mAb (anti-DRαβ dimers, IgG2a; BD Biosciences, Pont de Claix, France), H5C6 mAb (anti-CD63, IgG1; Developmental Studies Hybridoma Bank, The University of Iowa, Department of Biological Sciences, Iowa City, IA), unconjugated and FITC-conjugated H4A3 (anti-CD107a, IgG1; BD Biosciences), Cy5-conjugated F(ab′)2 donkey anti-goat IgGs, Cy3-conjugated F(ab′)2 donkey anti-mouse IgGs, Cy3 or Cy5-conjugated donkey anti-sheep IgGs (Jackson Immunoresearch, West Baltimore, PA) and Alexa 488-conjugated donkey anti-mouse or anti-goat IgGs (Invitrogen), all displaying minimal cross reactivity with mouse, rabbit, rat and human Igs. L243 and H5C6 were directly labeled using a Cy3 labeling kit (GE Healthcare) or Alexa 488 labeling kit (Invitrogen) according to the manufacturers' instructions.

Polyclonal rabbit anti-GFP antibodies (Invitrogen) were used for electron microscopy.

The 2.16.1 (IgG1) and 47.15.6 (IgG1) mAbs (anti-LAPTM5 cytoplasmic domain) were obtained by immunizing mice with a synthetic peptide (CMNSVEEKRNSKMLQKVVLPSYEEALSLPSKTPEGGPAPPPYSEV, corresponding to the last 45 amino acids of the cytoplasmic domain of LAPTM5) coupled to keyhole limpet hemocyanin (Biosynthesis, Lewisville, Texas, USA). Hybridomas were screened with an ELISA assay using a glutathione transferase-LAPTM5 cytoplasmic domain fusion protein expressed in *E. coli*.

### Biochemical analyses

Metabolic labeling and immunoprecipitations were performed as previously (Angenieux et al., 2000). Briefly, cells were metabolically labeled with [^35^S] methionine and cysteine. The labeled cells were resuspended in lysis buffer for 30 min on ice and the suspension was then cleared by centrifugation for 10 min at 13,000×g. CD1e proteins were immunoprecipitated and recovered onto protein A-Sepharose. The eluted proteins were treated or not with endoglycosidase H (Endo H) or PNGase F (Endo F) (New England Biolabs, Ipswich, MA) before separation by SDS-PAGE. The gels were fixed, dried and exposed for autoradiography.

For western blot analyses, the cleared lysates of transfected cells were incubated with the indicated antibodies, immune complexes were recovered by adsorption on protein A-Sepharose (GE Healthcare) and the eluted proteins were separated by SDS-PAGE and transferred to nitrocellulose membranes (Bio-Rad Laboratories, Marnes la Coquettte, France). Protein bands were identified using a West Pico chemiluminescent substrate (Thermo Fisher Scientific).

### Immunostaining and confocal microscopy

Cells, fixed and permeabilized for intracellular labeling, were stained as described previously (Angenieux et al., 2000) and examined under a Leica SP5 AOBS confocal microscope (Leica Microsystems).

### Image analysis

Protein colocalizations were quantified by analyzing stacks of confocal images obtained with similar acquisition parameters. In order to exclude strong fluorescent artifacts or saturating pixels and to select structures of interest, thresholds were determined automatically after data processing by wavelet transforms as already described [8], [11]. The colocalization of double labels was then estimated by intensity correlation analysis (ICA), basically as previously described [12] and using the dedicated ImageJ plugin (ICA), except that no particular areas of the images were selected by image segmentation. Quantitative analyses of coincident structures and ICA based on their relative intensities in the different channels were performed on the maximum intensity projections of the multiple labeled stacks and for various fields of view, in each experimental data set.

### Electron microscopy

Transfected cells expressing CD1e alone or co-expressing CD1e and EYFP-LAPTM5 were fixed and processed for cryoelectron microscopy as previously described (Angenieux et al., 2005). The cells were labeled with the anti-CD1e mAb 20.6 and then incubated with a rabbit anti-mouse bridging serum, followed by protein-A conjugated to 15 nm gold particles. After fixation, a polyclonal rabbit anti-GFP Ab was added and directly detected using protein-A conjugated to 10 nm gold particles.

### FRET/FLIM experiments in living cells

FLIM measurements were done by phase modulation in the frequency domain using a custom-made system based on a commercial module (Lifa, Lambert Instruments). The module was attached to a Leica DMIRBE inverted microscope equipped with a Coolsnap HQ CCD camera (Photometrics) and a 473 nm modulated laser diode (Omicron) used for excitation of the donor fluorophore. The laser light was coupled through an optical fiber to a spinning-disk module (CSU10, Yokogawa) fitted with a 473/561 dual-band dichroic filter (Semrock). Fluorescence emission was selected with a long-pass filter (LP510).

Prior to FLIM measurements, the cells were examined in the wide field using a mercury lamp with standard Leica filter cubes, in order to check the level of expression of the fluorescently tagged proteins (EYFP-LAPTM5 and CD1e-mCherry). The cells were kept in the culture medium and maintained at 37°C under 5% CO_2_ during all experiments. FLIM images were analyzed with LI-FLIM software (Lambert Instruments) to determine the mean fluorescence lifetime of each cell.

## Results

### LAPTM5 associates with CD1e in bafilomycin treated transfected M10 cells

**Figure 1:**
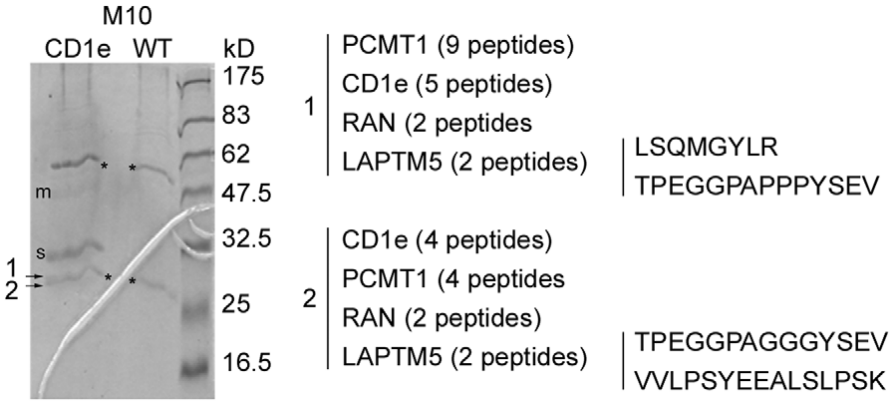
Identification of p27. Control (WT) and transfected M10 cells expressing CD1e (CD1e) were treated with bafilomycin and lyzed in detergent and CD1e proteins were immunoadsorbed on the immobilized mAb 20.6. Immune complexes were eluted, separated by SDS-PAGE and stained with Coomassie blue. Asterisks indicate the positions of the residual free Ig chains. In the two lanes, one mm sections were cut, digested with trypsin and analyzed by LC-MS/MS spectrometry. Analysis showed that bands m and s corresponded to membrane-associated and soluble CD1e molecules, respectively. The other peptides, also specific to the samples derived from M10CD1e cells, were only found in sections around the Ig light chain, at the expected position for p27, and are shown. The numbers of peptides derived from identified molecules are given, as are the amino acid sequences of the peptides assigned to LAPTM5.

**Figure 2:**
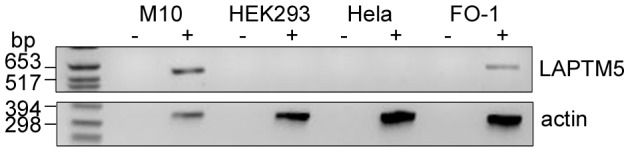
Melanoma cells express LAPTM5 transcripts. RNA samples from HeLa and HEK293 cells and from the melanoma cell lines M10 and FO-1 were analyzed by RT-PCR for the expression of LAPTM5; (−) control reaction without reverse transcriptase, (+) RT-PCR assays. Beta-actin- RT-PCR assays were performed for control experiments.

To identify p27, untransfected M10 cells and M10 cells expressing CD1e were treated for 5 hours with bafilomycin, clear lysates were prepared and CD1e molecules were immunoadsorbed on covalently-immobilized and cross-linked 20.6. The proteins were eluted and separated by SDS-PAGE. Coomassie blue staining (Fig. 1) revealed two bands at 47.5 kD and 32.5 kD, specific to the transfected M10 cell extracts and corresponding to membrane-associated and soluble CD1e respectively (bands m and s), in agreement with mass spectrometric analyses (data not shown and [10]). One mm sections were cut in the two lanes around the Coomassie-stained lower molecular mass species, corresponding to residual uncross-linked 20.6 light chains. After trypsin digestion, the sections were analyzed by LC/MS/MS spectrometry. Only two adjacent sections appeared to contain proteins specific to the sample derived from cells expressing CD1e. The same proteins were found in both sections, namely L-isoaspartate O-methyltransferase (encoded by *PCMT1*), CD1e, the GTP-binding nuclear protein Ran and lysosomal-associated transmembrane protein 5 (LAPTM5) (Fig. 1). Interestingly, LAPTM5 has been shown to accumulate in TGCs and to be addressed to the endosomal pathway. It recruits ubiquitin ligases, allowing its own ubiquitination and that of other partners such as GGA3 protein and subsequently its transport to lysosomes [13]. Thus, as LAPTM5 and CD1e molecules display a rather similar cellular distribution and are both ubiquitinated, their interaction would make sense. Since the expression of LAPTM5 is restricted to a limited number of cell types, we confirmed by RT-PCR that LAPTM5 is expressed in M10 cells and in another human melanoma cell line (FO-1) and checked that it is not expressed in HeLa and HEK293 human cell lines (Fig. 2).

### LAPTM5 and CD1e associate together under biological conditions

**Figure 3:**
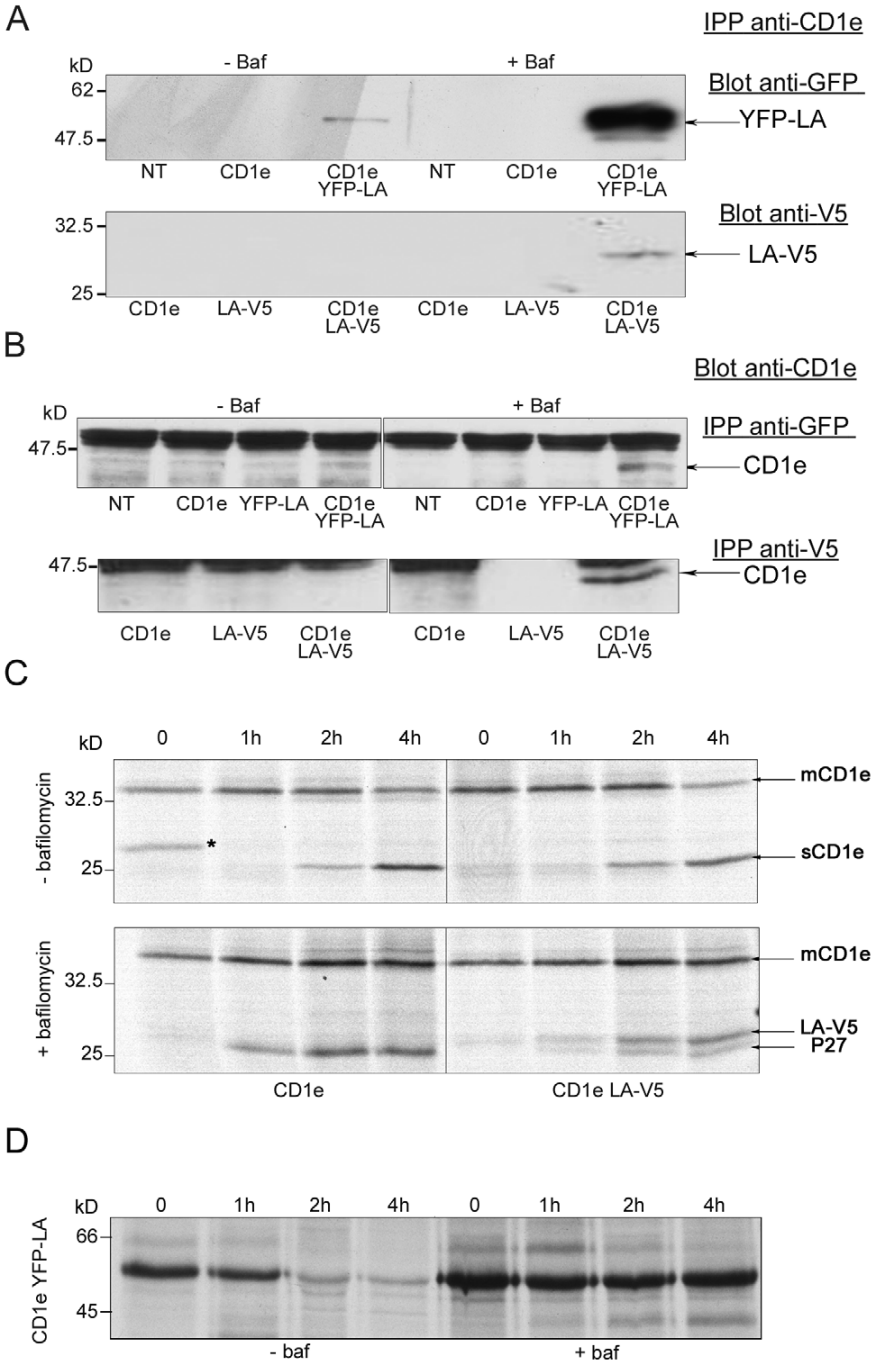
LAPTM5 co-immunoprecipitates with CD1e. Lysates of untransfected (NT) or transfected M10 cells expressing CD1e alone (CD1e), EYFP- or V5-tagged LAPTM5 alone (YFP-LA or LA-V5), or co-expressing CD1e and EYFP- or V5-tagged LAPTM5 (CD1e YFP-LA or CD1e LA-V5), untreated (−Baf) or treated (+Baf) with bafilomycin, were used in the following two experiments. A) Proteins were immunoprecipitated from cell lysates with the anti-CD1e mAb 20.6 and analyzed by western blotting using a rabbit anti-GFP Ab or an anti-V5 mAb. B) Cell lysates were immunoprecipitated with anti-GFP or anti-V5 Abs and analyzed by western blotting using the anti-CD1e mAb VIIC7. C) V5-tagged LAPTM5 competes with endogenous LAPTM5 for CD1e binding. Transfected M10 cells expressing CD1e alone (CD1e), or CD1e and LAPTM5-V5 (CD1e LA-V5), were metabolically labeled and chased in the absence or presence of bafilomycin. CD1e molecules were immunoprecipitated with the mAb 20.6, treated with Endo F and analyzed by SDS-PAGE. The asterisk in the left upper panel indicates a non specifically immunoadsorbed protein; mCD1e and sCD1e represent membrane-associated and soluble CD1e molecules, respectively. D) Bafilomycin protects EYFP-LAPTM5 from proteolysis. Transfected M10 cells expressing CD1e and the EYFP-LAPTM5 fusion protein were pulse chase labeled in the absence (−Baf) or presence (+Baf) of bafilomycin. Fusion proteins were immunoprecipitated with anti-GFP Abs.

To test whether LAPTM5 interacts with CD1e under the same conditions as p27 does, tagged LAPTM5 proteins were co-expressed with CD1e in M10 cells. The N-terminal and C-terminal ends of LAPTM5 are located on either side of the membrane in which it is inserted, the C-terminal end being in the cytosolic compartment. The C-terminal part carries sequences which control the cellular transport of LAPTM5, while no biological functions have yet been assigned to the short N-terminal sequence [13]. The LAPTM5 protein was fused with EYFP or V5 peptide tags at its N- or C-terminal end, respectively, and expressed in M10 cells expressing or not CD1e. The cells were treated or not for 5 hours with bafilomycin and lysed in detergent, after which immunoprecipitations and western blot analyses were performed. In a first series of experiments, CD1e molecules were immunoprecipitated with the mAb 20.6 and their association with LAPTM5 was confirmed by western blotting using anti-tag antibodies. As shown in the upper panel of Figure 3A, EYFP-LAPTM5 molecules co-immunoprecipitated with CD1e in untreated cells, and their association increased after bafilomycin treatment. These observations strongly suggest that LAPTM5 associates with CD1e, not only in bafilomycin-treated but also in untreated cells.

An interaction between LAPTM5-V5 and CD1e proteins could also be detected, but only after bafilomycin treatment (Fig. 3A, lower panel). In a second series of experiments, cleared lysates were immunoprecipitated with anti-tag antibodies (anti-GFP or anti-V5 Abs) and immunoblotted with the anti-CD1e mAb VIIC7. As seen in Figure 3B, the western blots of the proteins immunoprecipitated with anti-V5 or anti-GFP mAbs revealed the presence of CD1e molecules only when the cells had been treated with bafilomycin.

The association of LAPTM5-V5 with CD1e was then investigated in pulse-chase labeling experiments. M10 cells expressing CD1e alone or co-expressing CD1e and LAPTM5-V5 were metabolically labeled for 45 minutes with ^35^S and chased for 0, 1, 2 or 4 hours in the presence or not of bafilomycin. Membrane proteins were solubilized in 1% Triton X100 and incubated with the mAb 20.6. The immunoprecipitated proteins were deglycosylated with Endo F (Fig. 3C). In the absence of bafilomycin, the biochemical maturation of CD1e molecules was not affected by the expression of LAPTM5-V5, since the generation of soluble CD1e after 2 hours of chase was comparable, whether the cells co-expressed or not LAPTM5-V5. As expected, in the presence of bafilomycin, only membrane-associated CD1e molecules were metabolically labeled. The p27 and LAPTM5-V5 proteins both co-immunoprecipitated with CD1e and the electrophoretic mobilities of the two proteins were compatible with the hypothesis that p27 is LAPTM5. Interestingly, V5-tagged LAPTM5 molecules appeared to compete with endogenous p27 for CD1e binding. This competition was also observed in M10 cells co-expressing CD1e and EYFP-LAPTM5 (data not shown).

To elucidate why the interaction between CD1e and LAPTM5 is difficult to detect in the absence of bafilomycin, we checked the stability of EYFP-LAPTM5 in cells treated or not with bafilomycin. M10 cells co-expressing CD1e and EYFP-LAPTM5 were pulse-chase labeled in the presence or absence of bafilomycin and immunoprecipitated with anti-GFP mAbs. Autoradiography confirmed that in the absence of bafilomycin, EYFP-LAPTM5 molecules were rapidly proteolysed within the first two hours of chase. Conversely, in bafilomycin-treated cells no proteolysis was observed, even after four hours (Fig. 3D). This suggests that LAPTM5 has a relatively short half-life and that, like CD1e, it is processed by proteolytic enzymes acting in an acidic environment.

### CD1e and LAPTM5 colocalize in TGN and late endosomal compartments

**Figure 4:**
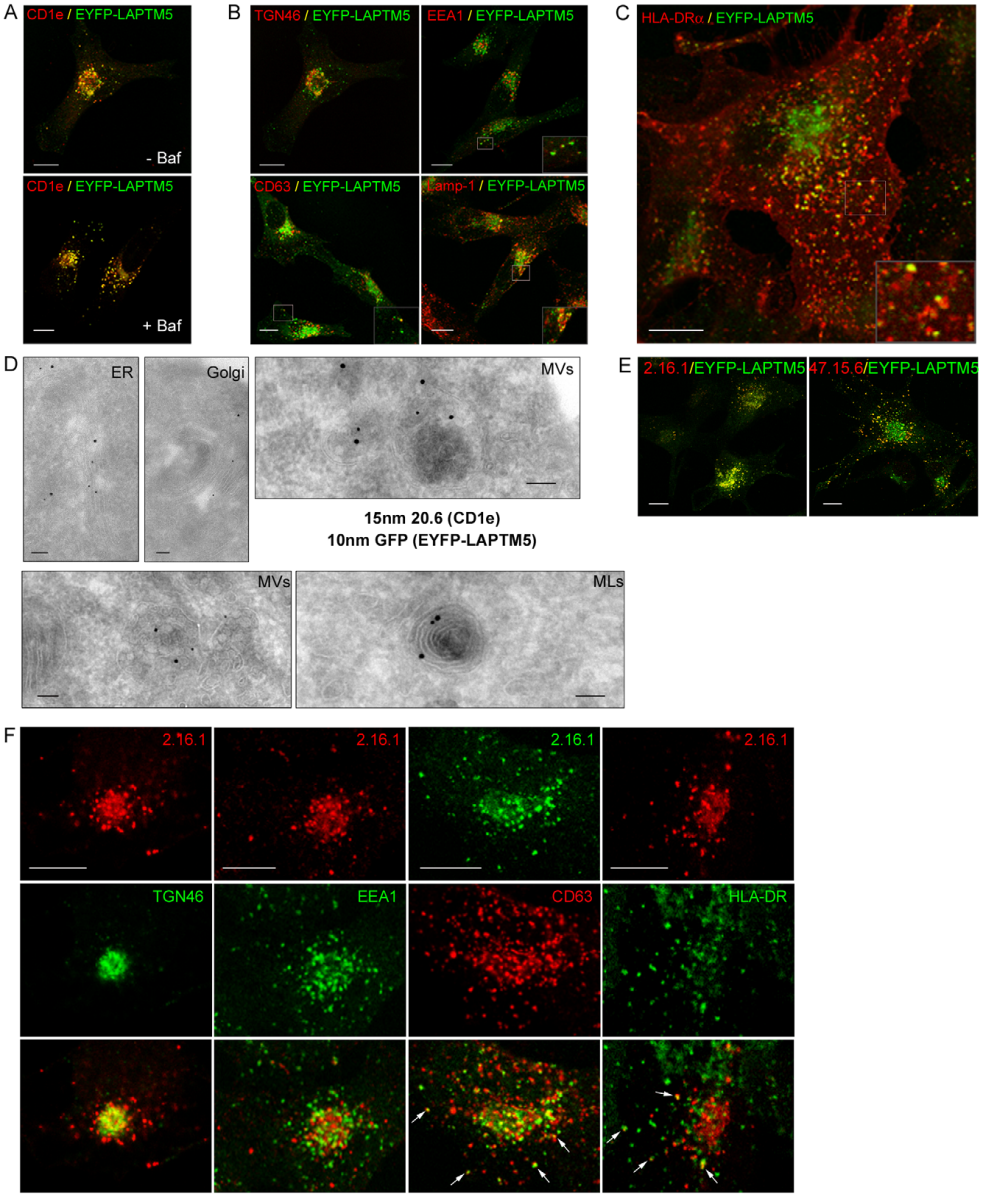
Cellular distribution of LAPTM5. Transfected M10 cells co-expressing EYFP-LAPTM5 and CD1e were fixed, permeabilized and co-stained with the anti-CD1e mAb 20.6, revealed using Cy3-conjugated antibodies (A) and antibodies specific for TGN46, revealed by Cy5-conjugated antibodies (B, left panel, represented using red pseudo-color), or stained with anti-EEA1, -CD63, -Lamp1 (B) or -HLA-DR Abs (C). Untreated cells (upper panel) were compared with cells treated for 4 hours with bafilomycin (lower panel) (A). D) M10 cells co-expressing CD1e and EYFP-LAPTM5 were fixed and processed for immunolabeling of cryosections with 10 nm anti-GFP and 15 nm anti-CD1e conjugated gold particles. EYFP-LAPTM5 and CD1e were detected in the endoplasmic reticulum (ER), in Golgi compartments (Golgi) and in multivesicular (MVs) and multilamellar endosomes (MLs). E, F) Characterization of two anti-LAPTM5 mAbs. M10 cells co-expressing CD1e and EYFP-LAPTM5 were fixed, permeabilized and stained with an anti-LAPTM5 mAb (2.16.1 or 47.15.6), which was revealed with Cy3-conjugated secondary Abs (E). M10 cells expressing CD1e were fixed, permeabilized and double-labeled with 2.16.1 and either anti-TGN46, anti-EEA1, anti-CD63 (H5C6) or anti-HLA-DR (L243) Abs (F). Scale bars, confocal micrographs, 10 µM; electron microscopy micrographs, 100 nM.

The cellular distribution of LAPTM5 was first analyzed by confocal microscopy in transfected M10 cells co-expressing CD1e and EYFP-LAPTM5. Analysis of fixed, permeabilized cells stained with the mAb 20.6 showed that CD1e and LAPTM5 colocalized in dense structures and vesicular structures, which were probably Golgi and endosomal compartments, respectively. After 4 hours of treatment with bafilomycin, the colocalization was almost total (Fig. 4A). Quantification of the colocalization confirmed the strong colocalization of CD1e and LAPTM5 molecules and showed that, locally within the cell, their respective amounts varied similarly (Fig. S1).

The identity of the EYFP-positive structures in untreated cells was confirmed by immunostaining with Abs specific for TGN-46 (Trans Golgi Network), EEA1 (sorting endosomes), CD63 (late endosomes) or Lamp-1 (CD107a, lysosomes). EYFP-LAPTM5 was present in dense TGN46^+^ Golgi structures and to a lesser extent in CD63^+^ and Lamp-1^+^ late compartments, while only juxtaposed to EEA1^+^ vesicles (Fig. 4B). Immunostaining with the mAb L243 (Fig. 4C) revealed that EYFP-LAPTM5 was present in HLA-DR^+^ compartments. This cellular distribution was also observed in transfected M10 cells expressing LAPTM5-V5, although some of these molecules appeared to be retained in the ER (data not shown).

The cellular colocalization of CD1e and LAPTM5 was confirmed by immunolabeling of cryosections of transfected M10 cells expressing CD1e alone or co-expressing CD1e and EYFP-LAPTM5. The cells expressing CD1e alone were stained by the anti-CD1e mAb, but not by the anti-GFP Abs (data not shown), showing that the immunodetection of EYFP-LAPTM5 in double transfected cells (Fig. 4D) was specific. Electron microscopy revealed CD1e and EYFP-LAPTM5 molecules in the endoplasmic reticulum (ER), Golgi compartments and multivesicular (MVs) and multilamellar endosomes (MLs).

Electron microscopy and confocal microscopy (Fig. S2A) confirmed that the expression of EYFP-tagged LAPTM5 did not significantly affect the cellular localization of CD1e in transfected M10 cells expressing endogenous LAPTM5. Similarly, in HEK293 cells, the expression of V5- or EYFP-tagged LAPTM5 did not modify the distribution of CD1e molecules (Fig. S2B and data not shown).

**Figure 5:**
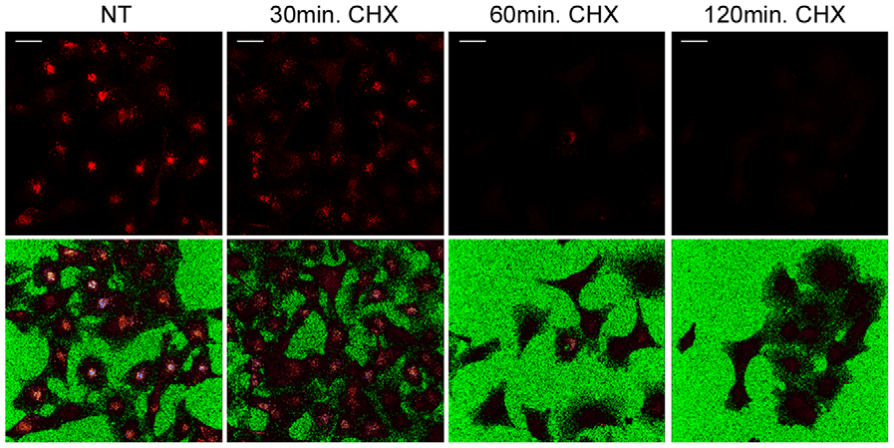
Short half life of LAPTM5. The turnover of LAPTM5 was investigated in cycloheximide-treated cells. M10 cells expressing CD1e were treated or not with cycloheximide 10 µg/ml (CHX). After fixation and permeabilization, the cells were labeled with 2.16.1 (upper row). The lower row reveals the positions of the cells. Scale bar: 30 µM.

To determine the efficiency of EYFP as an indicator of the localization of the fusion protein EYFP-LAPTM5 and the endogenous protein LAPTM5, two monoclonal antibodies specific for LAPTM5 were developed by immunizing mice with a synthetic peptide representing the entire C-terminal cytoplasmic tail. ELISA experiments using synthetic peptides corresponding to overlapping subsequences of the cytoplasmic tail showed that both mAbs bound to peptides containing the subsequence PSKTPEGGPA (data not shown). The specific recognition of LAPTM5 by these antibodies was confirmed by immunostaining transfected ^−^ HEK293 cells expressing tagged LAPTM5 (data not shown). Analysis of M10 cells expressing EYFP-LAPTM5 by confocal immunofluorescence microscopy showed that the mAb 2.16.1 staining fully overlapped the localization of EYFP, while the other mAb 47.15.6 only recognized EYFP^+^ vesicular compartments (Fig. 4E). The mAb 2.16.1 was thus used to check the cellular distribution of endogenous LAPTM5 in M10 cells expressing CD1e. Confocal immunofluorescence microscopy showed that endogenous LAPTM5, like the tagged LAPTM5 fusion proteins, colocalized with TGN-46, CD63 and HLA-DR. In contrast, only juxtaposition of LAPTM5^+^ and EEA1^+^ vesicles was observed (Fig. 4F). Use of these antibodies also allowed us to confirm that the half-life of LAPTM5 was rather short, since treatment for 60 min with cycloheximide resulted in a dramatic decrease in the immunolabeling of M10 cells (Fig. 5). Since LAPTM5 and EYFP-LAPTM5 displayed very similar colocalization patterns in M10 cells, we concluded that the observed strong colocalization of EYFP-LAPTM5 and CD1e was representative of that between endogenous LAPTM5 and CD1e.

**Figure 6:**
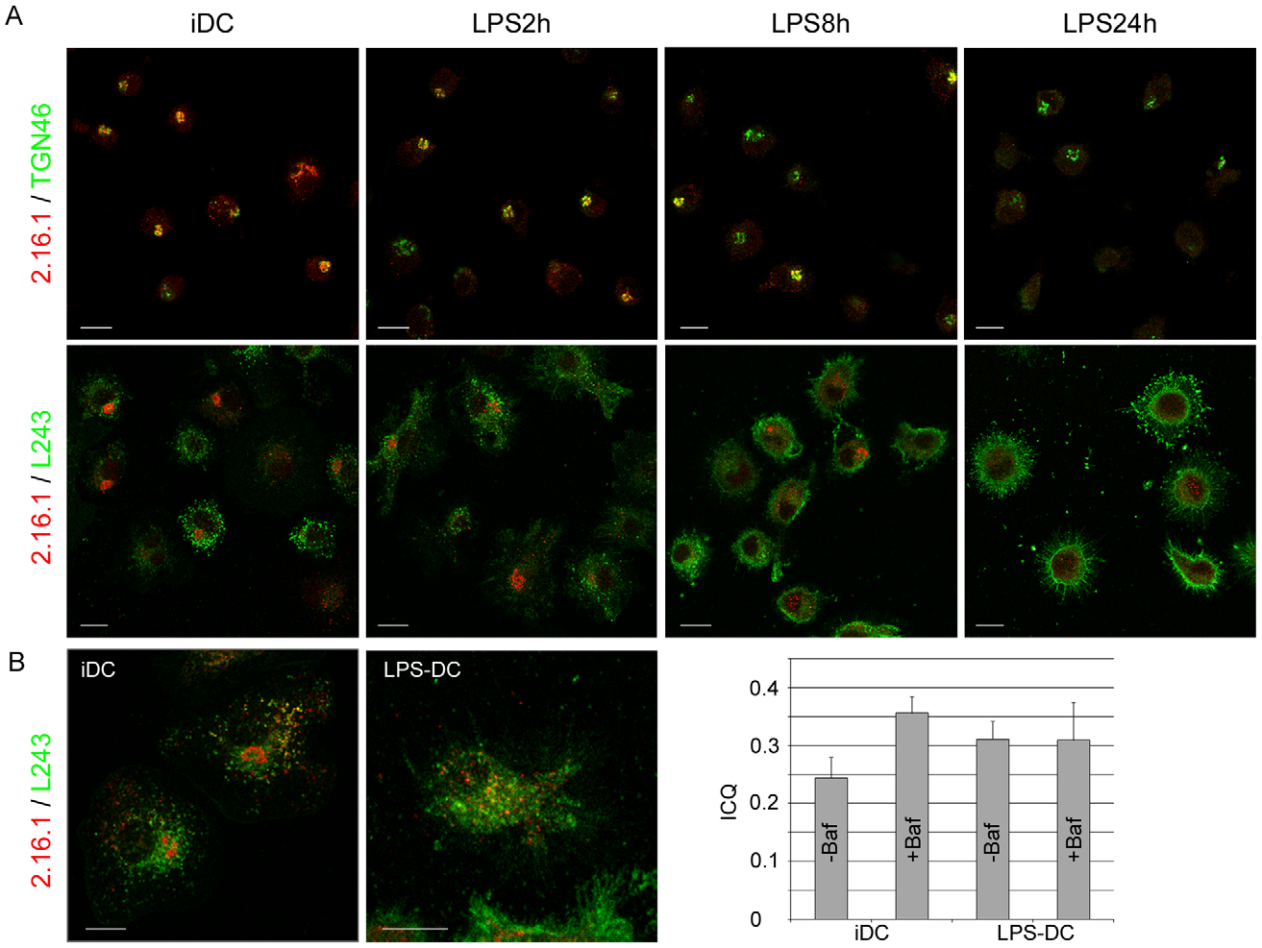
Localization of LAPTM5 in dendritic cells. A) Immature DCs and maturing DCs treated with LPS were allowed to adhere to glass coverslips precoated with poly-L-lysine. After fixation and permeabilization, the DCs were labeled with 2.16.1 and either anti-TGN46 or anti-HLA-DR (L243) Abs and analyzed by confocal microscopy. B) Immature DCs (iDC) and DCs treated with LPS for 4 hours (LPS-DC) were incubated for 1 hour with bafilomycin, before being allowed to adhere to glass coverslips. The cells were then fixed, permeabilized and double-labeled with 2.16.1 and anti-HLA-DR (L243). Scale bar: 10 µm. The intensity correlation quotient (ICQ) of cells treated (+Baf) or not (−Baf) with bafilomycin and stained with anti-LAPTM5 and anti-HLA-DR mAbs was calculated as described in the Methods section; the numbers of cells analyzed were 29 (iDC), 38 (iDC +Baf), 36 (LPS-DC) and 24 (LPS-DC +Baf).

Finally, we investigated the distribution of LAPTM5 in DCs derived from monocytes. In immature DCs, LAPTM5 colocalized most strongly with TGN-46, while after LPS-induced maturation, LAPTM5 became undetectable (Fig. 6A). The weak staining of LAPTM5 in the endosomes of immature and maturing DCs probably resulted from its rapid degradation in acidic endosomes, since treatment with bafilomycin allowed LAPTM5 to accumulate in these compartments (Fig. 6B). Calculation of the intensity correlation quotient (ICQ) [12] revealed that treatment for 4 hours with LPS resulted in an increased colocalization of LAPTM5 and HLA-DR molecules and in a simultaneous increase in the amounts of colocalized molecules. Moreover, while incubation with bafilomycin raised the ICQ in immature DCs, it had no major effect on this parameter in DCs treated for 4 hours with LPS, suggesting that the half-life of endosomal LAPTM5 increased under these latter conditions in LPS-treated cells (Fig. 6B, right). In contrast, the absence of LAPTM5 staining in fully mature DCs, obtained after 24 hours of treatment with LPS, could be explained by the down regulation of its gene, as revealed in RT-PCR experiments (data not shown). The cellular distributions of LAPTM5 and CD1e in immature dendritic cells are thus somewhat similar.

### CD1e and LAPTM5 physiologically interact in vivo

**Figure 7:**
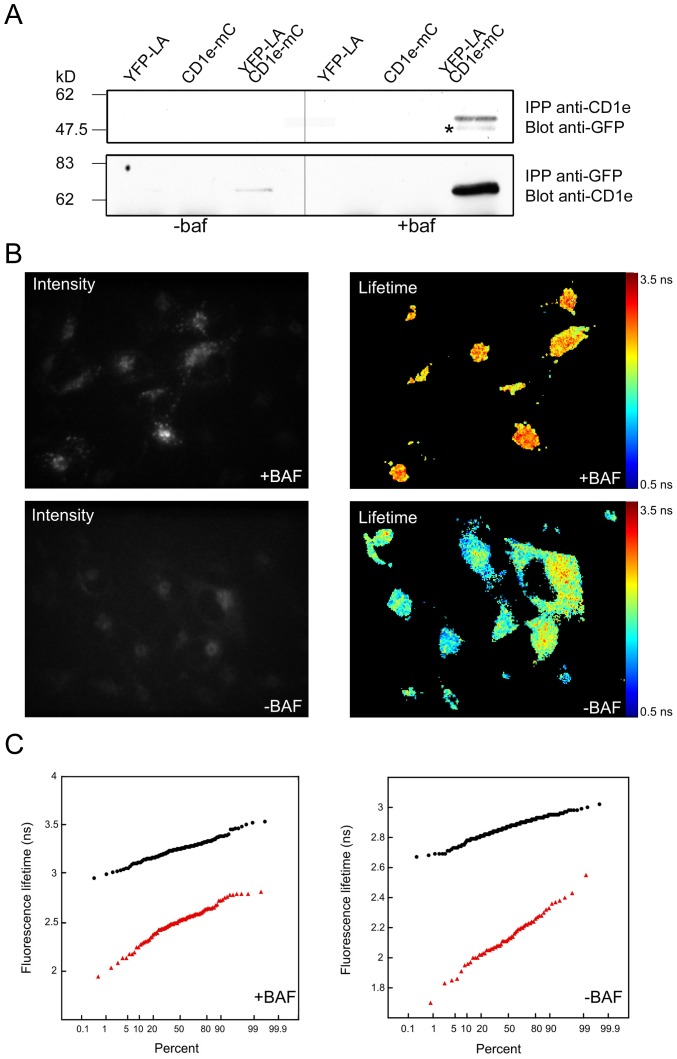
CD1e and LAPTM5 interact not only in bafilomycin treated cells but also in untreated cells. A) Co-immunoprecipitation of CD1e-mCherry and EYFP-LAPTM5. M10 cells expressing EYFP-LAPTM5 (YFP-LA) or CD1e-mCherry (CD1e-mC) alone, or co-expressing the two proteins, were treated or not with bafilomycin for 4 hours and lyzed in Triton X100. Proteins were immunoprecipitated using the mAb 20.6 or an anti-GFP Ab and analyzed by western blotting using anti-GFP or anti-CD1e (VIIC7) Abs respectively. The molecular species indicated by the asterisk is non specific or may derive from proteolysis of the full length fusion protein. B, C) Stably transfected M10 cells expressing EYFP-LAPTM5 or co-expressing CD1e-mCherry and EYFP-LAPTM5 were analyzed by FLIM in 37°C culture chambers. The fluorescence lifetimes of EYFP were measured by analyzing the indicated numbers of cells. B) Representative fluorescence lifetime acquisition in cells co-expressing CD1e-mCherry and EYFP-LAPTM5, after bafilomycin treatment (upper panels) or not (lower panels): intensity (left) and fluorescence lifetime of EYFP-LAPTM5 (right). C) Comparison of the fluorescence lifetimes of EYFP in singly (black dots) and doubly transfected cells (red triangles) using the probability plots for bafilomycin treated (left) or untreated cells (right).

CD1e-mCherry and EYFP-LAPTM5 fusion proteins were co-expressed in M10 cells to analyze the CD1e/LAPTM5 interaction *in vivo* by FRET/FLIM. Firstly, we checked whether the CD1e-mCherry fusion protein co-immunoprecipitated with EYFP-LAPTM5. M10 cells expressing CD1e-mCherry or EYFP-LAPTM5 alone, or co-expressing the two proteins, were treated or not for 5 hours with bafilomycin. Cleared lysates were immunoprecipitated with the 20.6 or an anti-GFP Ab and immunoblotted with an anti-GFP or the VIIC7 Ab, respectively (Fig. 7A). The western blot analyses showed that CD1e-mCherry and EYFP-LAPTM5 molecules co-immunoprecipitated and that their association increased after bafilomycin treatment.

FRET/FLIM experiments were performed in living M10 cells with or without bafilomycin treatment (Fig. 7B, C). As a control, the fluorescence lifetime was measured in cells expressing EYFP-LAPTM5 alone. In the presence of bafilomycin, the average fluorescence lifetime in cells expressing only EYFP-LAPTM5 was 3.23±0.10 ns, (N = 141), while the mean lifetime of EYFP in cells co-expressing CD1e-mCherry was 2.49±0.18 ns (N = 97) (Fig. 7C). The significant decrease in fluorescence lifetime indicated that energy transfer by FRET occurred. In order to assess the native association of the two proteins, we performed similar experiments in cells expressing EYFP-LAPTM5 and CD1e-mCherry in the absence of treatment with bafilomycin and obtained a mean fluorescence lifetime of 2.89±0.07 ns (N = 213) for cells expressing only EYFP-LAPTM5 and 2.13±0.16 ns (N = 62) for the doubly transfected cells (Fig. 7C). Once again, FRET occurred under these conditions, with very close values of the apparent transfer efficiency (∼0.23 for treated and ∼0.26 for untreated cells). The differences between the control and FRET situations were highly significant (p<0.0001). This is clearly visible on the probability charts (Fig. 7C), where 100% of the lifetime values of EYFP are shorter in EYFP-LAPTM5/CD1e-mCherry cells than in EYFP-LAPTM5 cells, whether the cells were treated or not with bafilomycin. These experiments showed that the molecules are in close proximity (about 10 nm) in living cells, in both bafilomycin treated and untreated cells. Thus, our biochemical and biophysical experiments supported the conclusion that the association of LAPTM5 with CD1e does not result from treatment of the cells with bafilomycin, but occurs endogenously in M10 cells.

### The generation of soluble CD1e does not depend on LAPTM5

**Figure 8:**
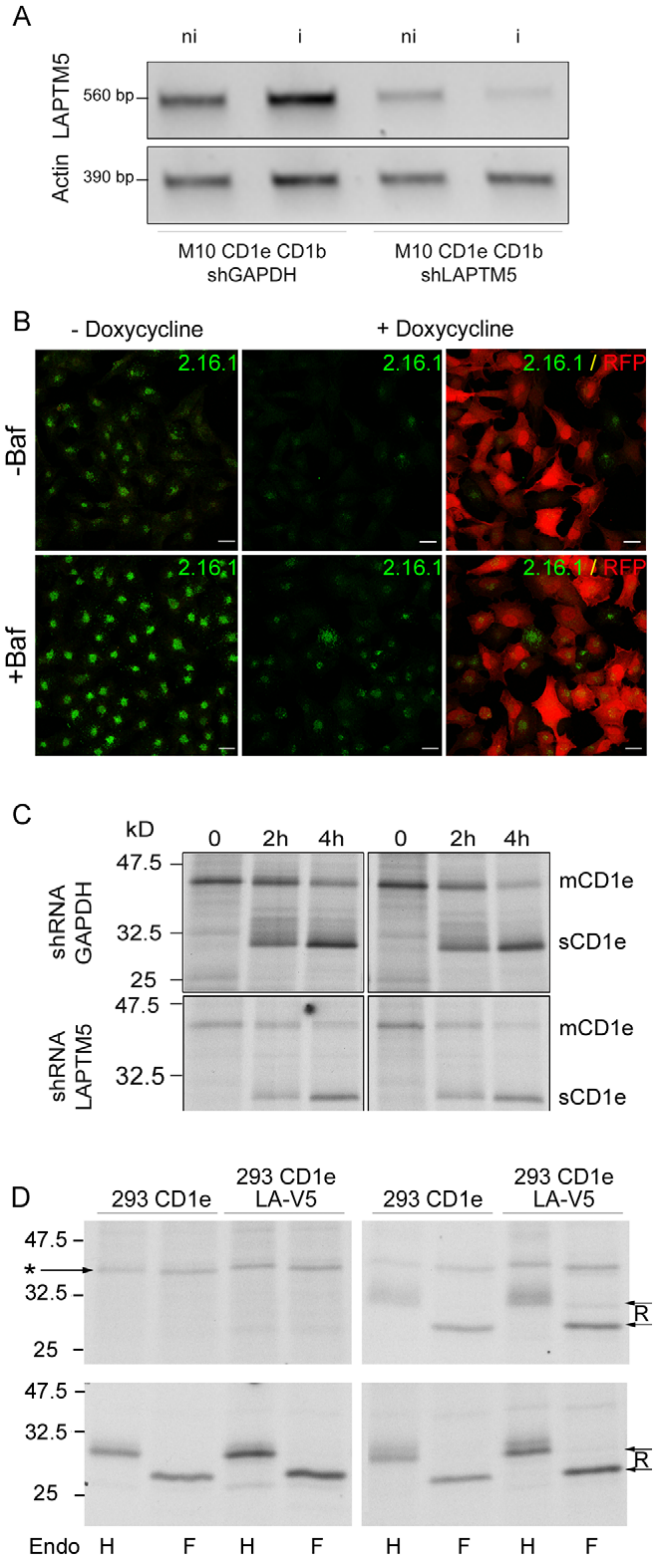
The generation of soluble CD1e is independent of LAPTM5 expression. A, B, C) M10 cells co-expressing CD1e and CD1b were transduced with lentiviral vectors expressing a doxycycline inducible anti-LAPTM5 shRNA or anti-GAPDH shRNA. A) The cells were cultured for 2 days in the absence (ni) or presence (i) of doxycycline (1 µg/mL). Knockdown of LAPTM5 expression was checked by RT-PCR (upper panel) and B) by immunofluorescence staining of fixed, permeabilized cells with the anti-LAPTM5 mAb 2.16.1, after treatment (+Baf) or not (−Baf) with bafilomycin. Scale bar: 20 µM. C) The cells were cultured for 2 days in the absence or presence of doxycycline and metabolically pulse chase labeled. CD1e molecules were immunoprecipitated with the mAb 20.6, treated with Endo F and analyzed by SDS-PAGE. mCD1e and sCD1e represent membrane-associated and soluble CD1e molecules, respectively. D) Transfected HEK293 cells expressing CD1e alone or co-expressing LAPTM5-V5 protein were metabolically labeled and chased for 0, 4, 8 and 18 hours. CD1e molecules were immunoprecipitated from Triton X100 lysates with the mAb 20.6, treated with Endo H or Endo F and analyzed by SDS-PAGE. The position of the Endo H-sensitive membrane-associated CD1e molecules is indicated by a star, those of the Endo H-resistant soluble CD1e molecules by arrows and “R".

To determine whether LAPTM5 affects the kinetics of the transport of CD1e molecules, we expressed a doxycycline-inducible LAPTM5 shRNA or a GAPDH control shRNA in M10 cells expressing CD1e. After 2 days culture in the presence of doxycycline to induce LAPTM5 knockdown, RT-PCR analysis showed that LAPTM5 extinction was efficient, although not complete (Fig. 8A), with 70% extinction of the expression of LAPTM5 after induction of the shRNA. Immunofluorescence with the mAb 2.16.1 confirmed this result (Fig. 8B). The biochemical maturation of CD1e after extinction of LAPTM5 was studied by pulse-chase labeling in the absence or presence of bafilomycin, followed by immunoprecipitation of the cleared lysates with the mAb 20.6. Autoradiography indicated that the extinction of endogenous LAPTM5 did not significantly affect the kinetics of maturation of membrane-associated CD1e into a soluble form (Fig. 8C). Overnight chase conditions further confirmed that the stability of soluble CD1e was not affected.

This absence of a noticeable effect of LAPTM5 on the maturation of CD1e molecules was confirmed again in a transfected HEK293 cell line. Control experiments were first performed to check that CD1e and LAPTM5-V5 co-immunoprecipitated in transfected HEK293 cells treated with bafilomycin (data not shown). Nevertheless, autoradiography revealed that the expression of LAPTM5 in transfected HEK293 cells did not significantly modulate the kinetics of maturation of membrane-associated CD1e into its soluble form or the fate of sCD1e molecules (Fig. 8D).

### Ubiquitination of CD1e does not depend on LAPTM5

We previously showed that ubiquitination of the cytoplasmic tail of CD1e appears to trigger its exit from Golgi compartments and its transport to endosomes [5]. Hence we used the LAPTM5 deficient HEK293 cell line to study the impact of LAPTM5 on the ubiquitination of CD1e. HEK293 cells expressing CD1e or LAPTM5-V5 alone, or co-expressing both molecules, were treated or not for four hours with bafilomycin. The CD1e proteins from cleared lysates were immunoprecipitated with the mAb 20.6 and immunoblotted with anti-ubiquitin or VIIC7 Abs (Fig. S3). The analysis revealed that CD1e was ubiquitinated in untreated cells and in greater amounts after treatment with bafilomycin. Essentially three CD1e species could be specifically identified and these had electrophoretic mobilities compatible with the coupling of one, two or three ubiquitins. Disappointingly, the co-expression of LAPTM5-V5 had no significant effect on the ubiquitination profile of CD1e.

## Discussion

The aim of this work was to identify the 27 kD protein which was found to associate with CD1e during its transport to late endosomal compartments when the cells are treated with bafilomycin, a selective inhibitor of the vacuolar H-ATPases responsible for endosome acidification. When CD1e was immunopurified from solubilized extracts of bafilomycin treated transfected cells, three proteins having the expected electrophoretic mobility co-purified, namely LAPTM5, L-isoaspartate O-methyltransferase (PCMT1) and the GTP-binding nuclear protein Ran. Since PCMT1 is a nuclear and cytoplasmic protein [14] involved in protein repair, while Ran is nuclear protein [15], we did not consider these two proteins to be good candidates for p27. In contrast, a biological meaning for the association of CD1e with LAPTM5 was suggested by a number of facts. Firstly, it is known that mouse LAPTM5 accumulates in TGN compartments and then is directly transported to the endosomal network, where it reaches late endosomal compartments; LAPTM5 and CD1e thus appear to have similar cellular distributions. Secondly, the exit of mouse LAPTM5 from the TGN and its transport to late endosomes is dependent on the consecutive recruitment of the ubiquitin ligase NEDD4 and the adaptor protein GGA3. More recently, ITCH ubiquitin ligase has been shown to regulate the transport and the fate of LAPTM5 in lysosomes [16]. Thirdly, the mouse and human LAPTM5 proteins have been shown to modulate a number of immunological pathways through the down-regulation of signaling molecules, probably by mediating their ubiquitination and subsequent degradation [17], [18], [19]. Since monoubiquitination of CD1e mediates its transport from the TGN to endosomes, its interaction with LAPTM5 would make sense.

Originally, the expression of LAPTM5 was thought to be restricted to the hematopoietic lineage and embryonic stem cells [20]. Later it was found to be up-regulated in glial cells in response to the apoptosis of neurons [21] and more recently, to be expressed in benign neuroblastomas [22]. We demonstrate here that LAPTM5 is also expressed in two melanoma cell lines. Transcriptome analyses indeed suggest that LAPTM5 is significantly expressed in some but not all melanomas, while poorly in melanocytes (see Geoprofile, Reference series GSE4570) [23]. How the expression of LAPTM5 in melanoma transformation is regulated and how it affects its malignancy would thus be worthwhile investigating.

We showed that CD1e and LAPTM5 molecules are present in the same trans-Golgi and endosomal compartments of transfected cells. We confirmed that CD1e and LAPTM5 do interact with one another using (i) co-immunoprecipitation followed by western blot analyses, (ii) pulse chase labeling followed by immunoprecipitation and (iii) FRET/FLIM experiments, using CD1e and LAPTM5 tagged with the fluorescent proteins mCherry and EYFP, respectively. Very importantly, this interaction occurred not only in bafilomycin treated cells but also under physiological conditions, when the cells were not treated. However, in transfected cells, the biochemical maturation and cellular transport of CD1e appeared not to be significantly influenced by this interaction.

In immature human dendritic cells, LAPTM5 fully colocalized with TGN46, where CD1e molecules are also present. LAPTM5 was not detected in endosomes, probably because it is rapidly degraded in these compartments, as observed in our M10 cell line model. Consistent with this view, when immature DCs were treated with bafilomycin, LAPTM5 accumulated in CD63^+^ late endosomal compartments, as reported for CD1e molecules. We observed that in mature DCs, LAPTM5 was down regulated, as documented for CD1e. However, while the maturation of DCs resulted in an increased stability of lysosomal CD1e molecules and their accumulation in lysosomes [8], LAPTM5 was no longer detectable. This would suggest that it has inhibitory functions involving immunological processes in immature DCs, in relation to their tolerogenic activity. The use of mouse models knocked down for LAPTM5 [18], and/or transgenic for CD1e [4] and other human CD1 genes [24], might make possible to investigate the immunological impact of CD1e/LAPTM5 interaction on the presentation of CD1-restricted antigens.

## Supporting Information

Figure S1 **We used the image correlation analysis (ICA) method to test for a staining relationship between LAPTM5 (upper left) and CD1e (upper right) in M10 cells (see** **Methods** **).** Note that the plots (middle panels) were strongly skewed towards positive values, consistent with a highly dependent staining pattern. On the basis of these values, we created a correlative image (lower left) and a frequency scatter plot indicating the relative intensities of the pixels in channel 1 for LAPTM5 (X axis) and in channel 2 for CD1e (Y axis) (lower right). This shows both the coincidence of double stained pixels and the variations in the intensities in the two channels in one cell (see arrows in the images and in the frequency scatter plot). Furthermore, the calculated ICQ values in 14 distinct fields of view were consistently positive and highly significant (+0.314±0.05, p<0.001, n = 14). This analysis provides compelling evidence that CD1e and LAPTM5 molecules not only colocalize but also display parallel local variations in their numbers.
(TIF)

Figure S2 **Over-expression of LAPTM5 does not affect the cellular distribution of CD1e molecules.** A) Fixed, permeabilized M10 cells expressing CD1e alone or co-expressing CD1e and EYFP-LAPTM5 were stained with the anti-CD1e mAb 20.6 and antibodies specific for TGN46, EEA1, CD63 or HLA-DR. B) Transfected HEK293 cells expressing CD1e alone or co-expressing LAPTM5-V5 were fixed, permeabilized and stained with the anti-CD1e mAb 20.6 and antibodies specific for TGN46, EEA1 and CD63. Scale bar, 10 µM.
(TIF)

Figure S3 **The ubiquitination of CD1e does not depend on LAPTM5.** Transfected HEK293 cells expressing CD1e (CD1e) or V5-tagged LAPTM5 (LA-V5) alone, or co-expressing CD1e and V5-tagged LAPTM5 (CD1e LA-V5), were treated (+) or not (−) with bafilomycin. CD1e molecules were immunoprecipitated with the mAb 20.6 and analyzed by western blotting using an HRP-conjugated anti-ubiquitin mAb or the anti-CD1e mAb VIIC7.
(TIF)
