# Supplementary figures and images for: Lysosomal-Associated Transmembrane Protein 5 (LAPTM5) Is a Molecular Partner of CD1e
Source: PLoS One. 2012 Aug 3;7(8):e42634. doi: 10.1371/journal.pone.0042634 (PMC3411835; doi:10.1371/journal.pone.0042634)

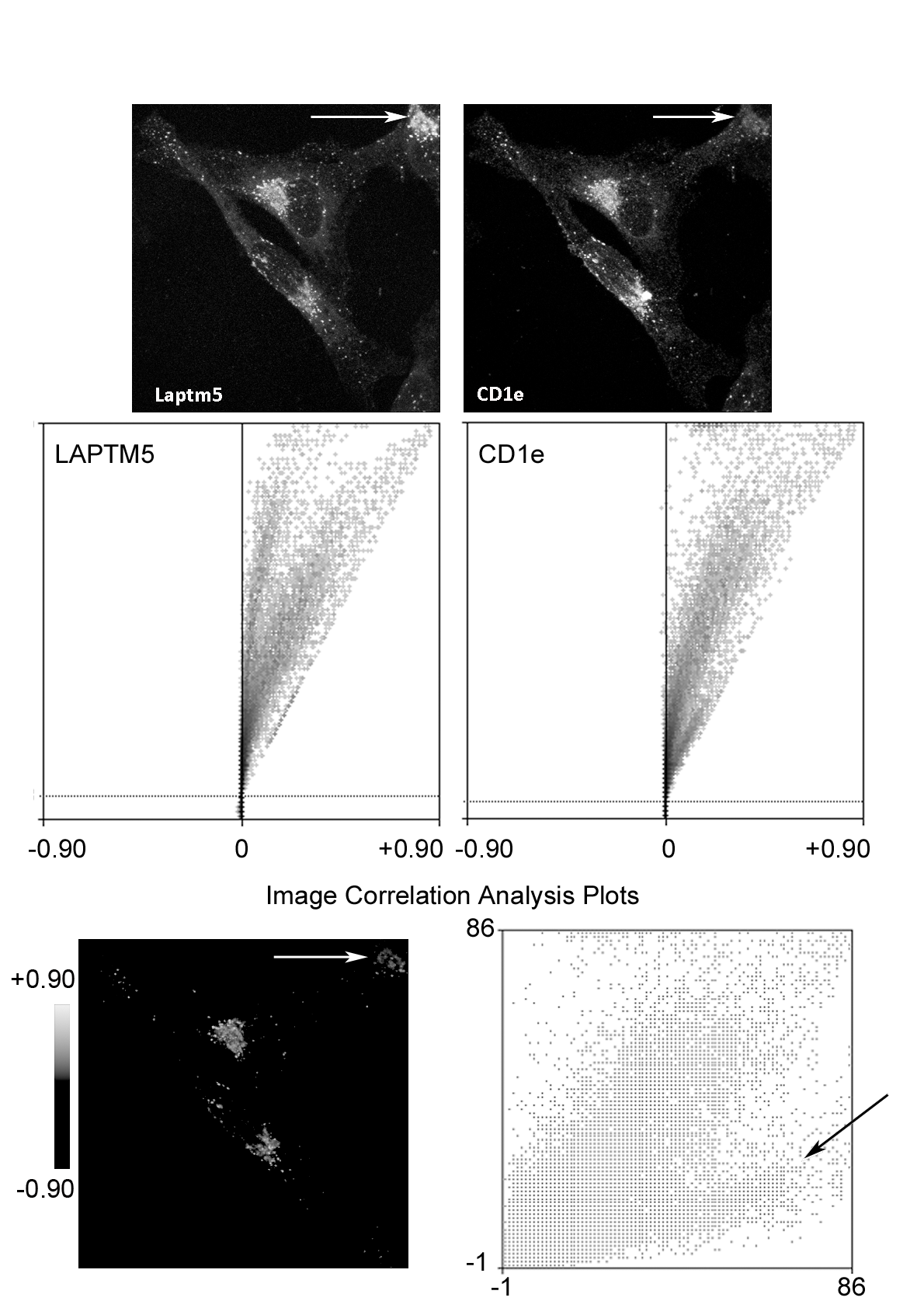

Supplement: Figure S1 — We used the image correlation analysis (ICA) method to test for a staining relationship between LAPTM5 (upper left) and CD1e (upper right) in M10 cells (see Methods ). Note that the plots (middle panels) were strongly skewed towards positive values, consistent with a highly dependent staining pattern. On the basis of these values, we created a correlative image (lower left) and a frequency scatter plot indicating the relative intensities of the pixels in channel 1 for LAPTM5 (X axis) and in channel 2 for CD1e (Y axis) (lower right). This shows both the coincidence of double stained pixels and the variations in the intensities in the two channels in one cell (see arrows in the images and in the frequency scatter plot). Furthermore, the calculated ICQ values in 14 distinct fields of view were consistently positive and highly significant (+0.314±0.05, p<0.001, n = 14). This analysis provides compelling evidence that CD1e and LAPTM5 molecules not only colocalize but also display parallel local variations in their numbers. (TIF) [file pone.0042634.s001.tif]

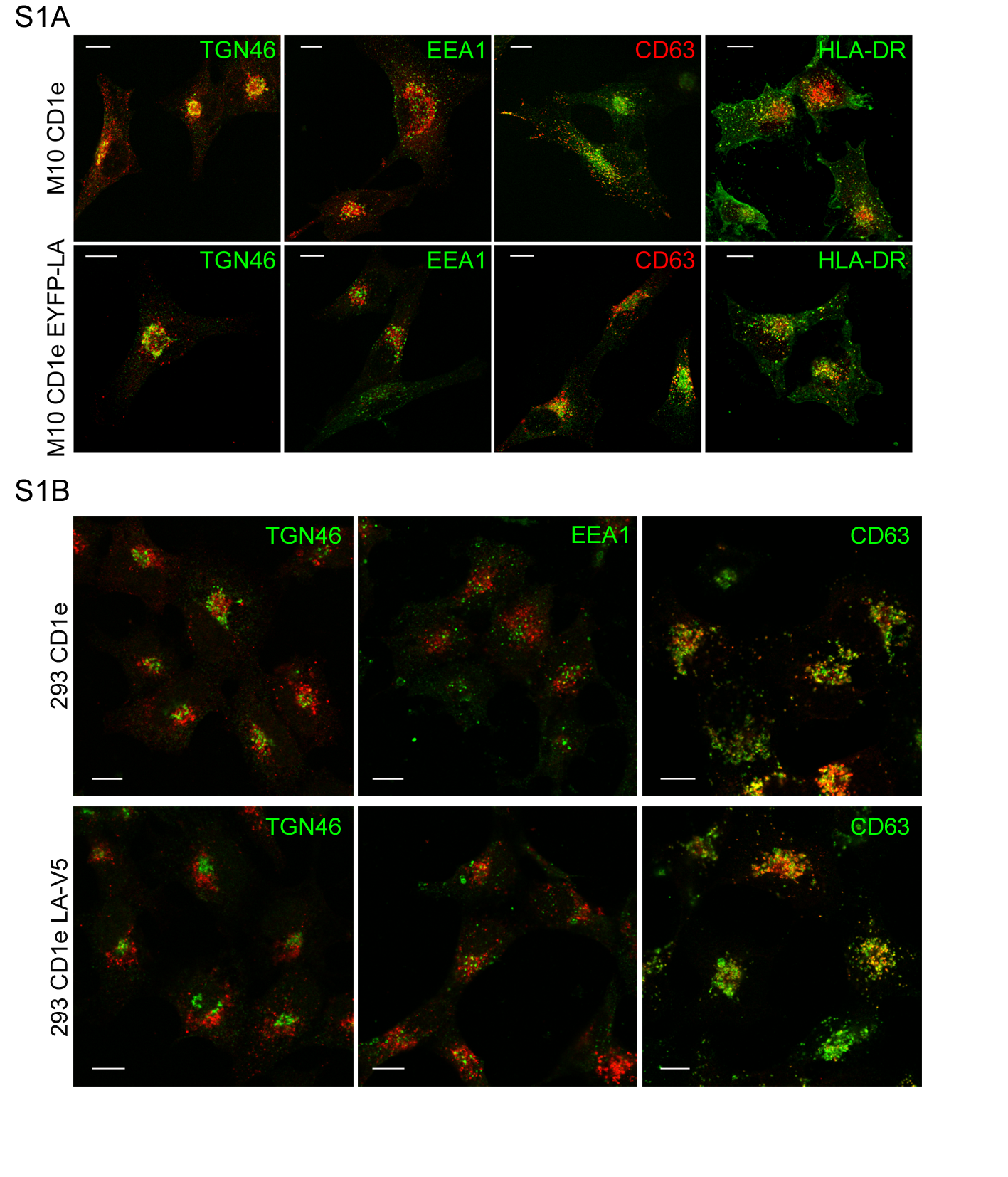

Supplement: Figure S2 — Over-expression of LAPTM5 does not affect the cellular distribution of CD1e molecules. A) Fixed, permeabilized M10 cells expressing CD1e alone or co-expressing CD1e and EYFP-LAPTM5 were stained with the anti-CD1e mAb 20.6 and antibodies specific for TGN46, EEA1, CD63 or HLA-DR. B) Transfected HEK293 cells expressing CD1e alone or co-expressing LAPTM5-V5 were fixed, permeabilized and stained with the anti-CD1e mAb 20.6 and antibodies specific for TGN46, EEA1 and CD63. Scale bar, 10 µM. (TIF) [file pone.0042634.s002.tif]

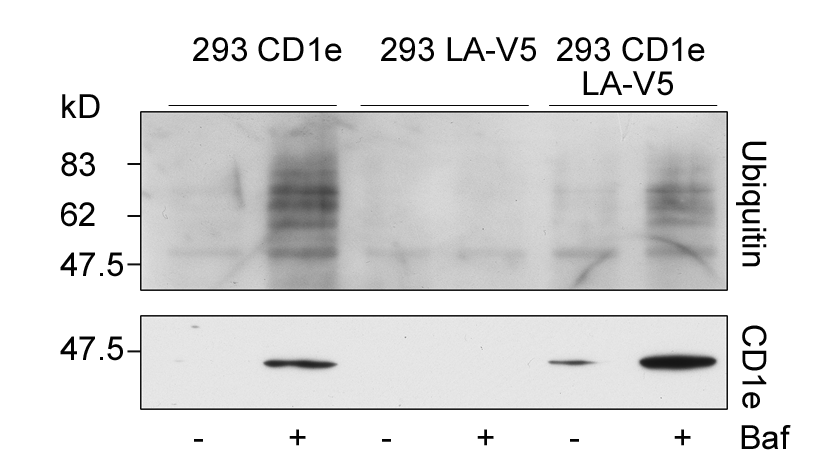

Supplement: Figure S3 — The ubiquitination of CD1e does not depend on LAPTM5. Transfected HEK293 cells expressing CD1e (CD1e) or V5-tagged LAPTM5 (LA-V5) alone, or co-expressing CD1e and V5-tagged LAPTM5 (CD1e LA-V5), were treated (+) or not (−) with bafilomycin. CD1e molecules were immunoprecipitated with the mAb 20.6 and analyzed by western blotting using an HRP-conjugated anti-ubiquitin mAb or the anti-CD1e mAb VIIC7. (TIF) [file pone.0042634.s003.tif]
